# Education on medically unexplained symptoms: a systematic review with a focus on cultural diversity and migrants

**DOI:** 10.1186/s40001-023-01105-7

**Published:** 2023-04-03

**Authors:** An Mariman, Peter Vermeir, Marta Csabai, Anne Weiland, Karen Stegers-Jager, Ruben Vermeir, Dirk Vogelaers

**Affiliations:** 1 Faculty of Medicine and Healthcare Sciences, Ghent University, Ghent, Belgium; 2 Centre for Integrative Medicine, Ghent University Hospital, Ghent, Belgium; 3 Dean’s Office of the Faculty of Medicine and Healthcare Sciences, Ghent University Hospital, Corneel Heymanslaan 10, 9000 Ghent, Belgium; 4 Institute of Psychology, Károli Gáspár University of the Reformed Church, Budapest, Hungary; 5 Department of General Practice, Erasmus MC University Medical Center, Rotterdam, The Netherlands; 6 Department of Internal Medicine, Erasmus MC University Medical Center, Rotterdam, The Netherlands; 7 Institute of Medical Education Research, Erasmus MC University Medical Center, Rotterdam, The Netherlands; 8 Department of Internal Medicine and Pediatrics, Ghent University Hospital, Ghent, Belgium; 9 Department of General Internal Medicine, AZ Delta, Roeselare, Belgium

**Keywords:** Medical unexplained symptoms (MUS), Somatoform disorder, Functional syndrome, Diversity, Migrants, Ethnicity, Care models, Medical education, Communication skills, Health literacy

## Abstract

**Background:**

Health care providers often struggle with the management of patients with medically unexplained symptoms (MUS), especially in case of a different ethnicity and/or cultural background. These challenges are insufficiently addressed in their training.

**Objectives:**

A systematic review on education in the field of MUS in a diverse context to improve MUS healthcare provider–patient interaction focused on intercultural communication.

**Methods:**

Screening of PubMed, Web of Science, Cinahl and Cochrane Library on the keywords ‘Medical unexplained (physical) symptoms (MUS)’, ‘Somatoform disorder’, ‘Functional syndrome’, ‘Diversity’, ‘Migrants’, ‘Ethnicity’, ‘Care models’, ‘Medical education’, ‘Communication skills’, ‘Health literacy’.

**Results:**

MUS patients, especially with a different ethnic background, often feel not understood or neglected. Health care providers experience feelings of helplessness, which may provoke medical shopping and resource consumption. Attitudes and perceptions from undergraduate trainees to senior physicians tend to be negative, impacting on the quality of the patient/health care provider relationship and subsequently on health outcomes, patient satisfaction and therapeutic adherence. Current undergraduate, graduate and postgraduate education and training does not prepare health care providers for diagnosing and managing MUS patients in a diverse context. A continuum of training is necessary to achieve a long term and lasting change in attitudes towards these patients and trainers play a key role in this process. Hence, education should pay attention to MUS, requiring a specific competency profile and training, taken into account the variety in patients’ cultural backgrounds.

**Conclusions:**

This systematic review identified significant gaps and shortcomings in education on MUS in a diverse context. These need to be addressed to improve outcomes.

## Key messages

- Current dominantly biomedical thinking hampers adequate management.
- A continuum of education and training focusing on a biopsychical approach is largely lacking and should be enhanced.
- Increasing competencies in intercultural communication in the different levels of health care is needed to address the additional complexity of MUS management in a context of increasing diversity.
- In view of the high prevalence of MUS and the obvious gaps detected, research and dedication of health care resources should be intensified.

## Introduction

Offering an acceptable explanatory model to patients with medically unexplained symptoms (MUS) remains a challenge, especially due to controversy on etiology and pathophysiology. Different theoretical explanatory frameworks have been validated only to a limited extent, and these, in general, integrate biological, psychological and social factors (e.g., in the biopsychosocial model, the stress–vulnerability model, the stress model, the perceptual–cognitive model, the neurobiological model, vicious circles and emotions) [1–4]. Even with an acceptable explanatory model, the quality of communication will determine the health care provider–patient relationship, and, in MUS, both this quality of communication and this relationship have a positive impact on health outcomes, patient satisfaction and therapeutic adherence [5, 6].

Difficulties in communication and appropriate approach are even greater in patients with a different ethnic background. In an increasingly multicultural and diverse society, patients with different racial and ethnic backgrounds and refugees experience disparities in access to qualitative healthcare [7].

These challenges are insufficiently addressed both in graduate and postgraduate training of health care professionals. To improve healthcare provider–patient interaction in these domains we performed a systematic review of the literature on education on MUS in the setting of increasing diversity to define gaps as well as areas for improvement and derive recommendations.

## Materials and methods

A systematic search was conducted on the databases PubMed, Web of Science, Cinahl and The Cochrane Library using the keywords: ‘Medically unexplained (physical) symptoms (MUS),’ ‘Somatoform disorder’, ‘Functional syndrome’, ‘Diversity’, ‘Migrants’, ‘Ethnicity’, ‘Care models’, ‘Medical education’, ‘Communication skills’, ‘Health literacy’. The keywords were internally validated by the co-authors. To qualify articles needed to be (1) published between January 1, 2002, and September 30, 2019; (2) available as full text in English; (3) categorizable as original research, reviews, meta-analyses or letters to the editor. Database screening was closed 31st of May 2021. Titles and abstracts were reviewed to verify inclusion criteria. If all inclusion criteria were present or if this remained unclear, the articles were fully read. All studies were screened for eligibility by two independent reviewers (PV, AM) who reviewed titles, abstracts, and full text. All disagreements were resolved by discussion and, if necessary, a third reviewer (DV) was consulted. Additional literature was obtained through searching references in the manuscripts (snowball method).

**Fig. 1:**
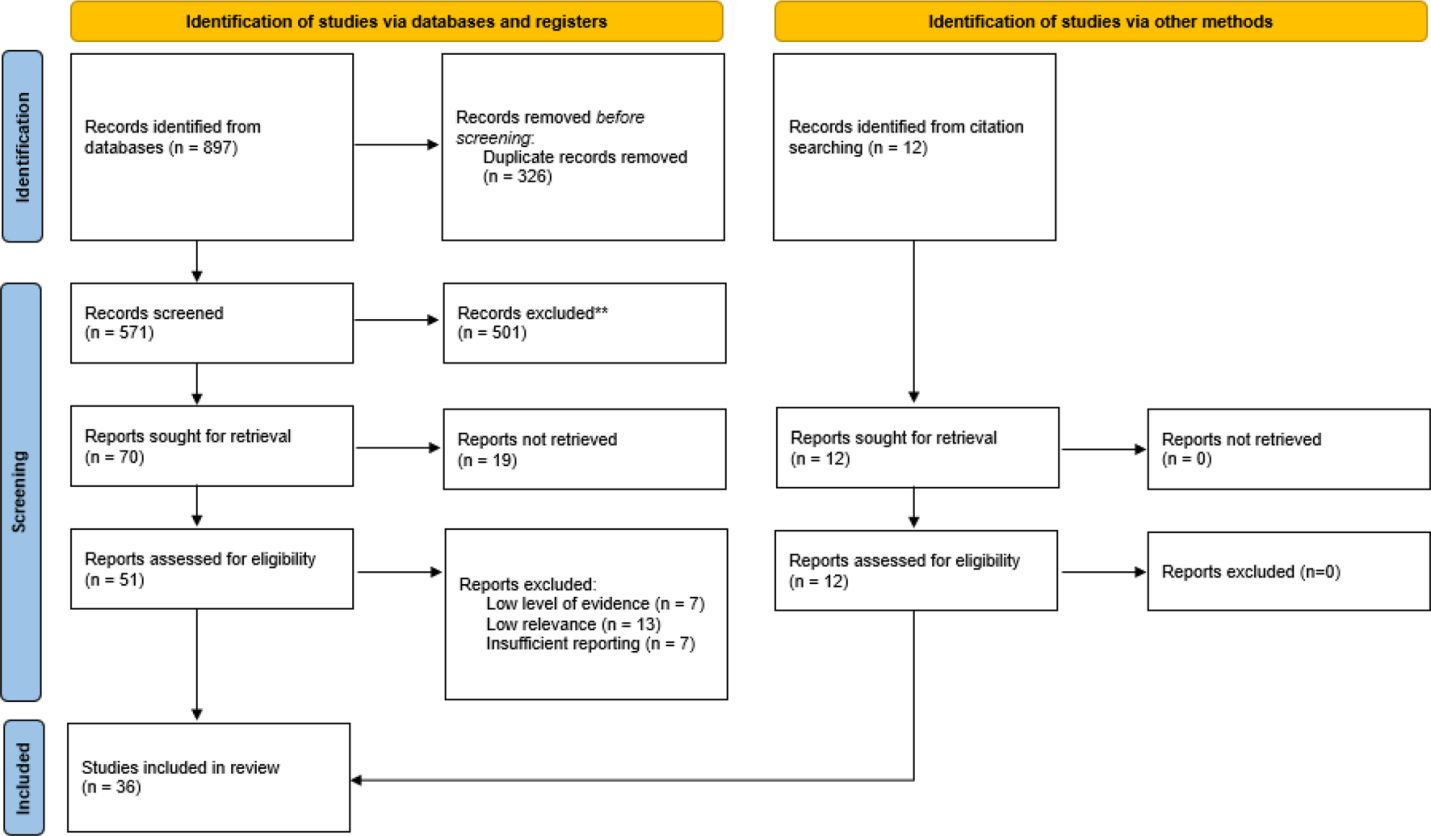
Review stages based on PRISMA flow diagram

The results of the search process are summarized into a PRISMA flow diagram (Fig. 1) [8]. Out of a total of 909 papers selected, 326 duplicates were removed.

After screening 583 papers on title and abstract 63 papers remained for full-text screening. From these, 36 articles were subjected to quality assessment [9].

## Results

MUS or functional syndromes are largely absent from the curricula of undergraduate medical training [10]. This lack of specific undergraduate training was also underpinned in semi-structured interviews of UK medical students in their 3^rd^ year of training. Despite discussing many matters surrounding chronic fatigue syndrome/myalgic encephalomyelitis (CFS/ME), all participants reported having received no formal CFS/ME training to date. Some felt that CFS/ME was not included, because the condition was too controversial, complex and unclear; nevertheless, the students indicated they would find training to be beneficial in their future roles as physicians. Despite this, CFS/ME was viewed as psychosocial or psychosomatic and, therefore, perceived of lower priority than other illnesses [11]. In interviews with medical educators from UK medical schools, different barriers for dedicating time in the curriculum to teaching about “unexplained symptoms” were identified, such as low priority in competition for teaching time or negative attitudes of educators towards functional syndromes. This reflects a very low status of “unexplained symptoms” in the prestige hierarchy of diseases among physicians and medical students as well as the perception of complexity as difficult to handle [12–14]. The tendency to look for single explanations instead of recognizing the broader context of complexity seems strongly embedded in both societal and educational thinking [15].

Undergraduate and graduate training fully focuses on biomedical disease perspectives. There is an apparent discrepancy between the ideal of diagnosable and curable diseases with biological causes and objective findings, learned during medical training, and the reality encountered in practice of people suffering from illness and social distress, presenting with subjective symptoms and need of care. This is evidenced by semi-structured interviews of Swedish general practitioners (GPs) and specialists from different fields experienced with MUS patients. This discrepancy may challenge the physician role model and lead to skepticism about conditions, such as CFS and fibromyalgia. Conditions regarded as illness in the absence of established pathogenetic mechanisms and a simple cause–effect relationship were considered as less serious than those with recognized disease status with established pathogenesis. Moreover, it was shown that these patient groups, in particular with fatigue and severe disability, do not always gain full access to the sick role or recognition of the impact on their disability, in part as a consequence of the conditions not being defined as diseases [16].

In qualitative in-depth interviews junior doctors in the UK identified a significant gap in their training on the topic of MUS, particularly in their awareness of this topic, the appropriate level of investigations, possible psychological comorbidities, the formulation of suitable explanations for patients’ symptoms and longer term management strategies. Many junior doctors expressed feelings of anxiety, frustration, and a self-perceived lack of competency in this area. They indicated over-investigating patients or avoiding patient contact altogether due to the challenging nature of MUS and experienced difficulty in managing the accompanying uncertainty. They reported feeling helpless and unsure about their role in patient management and thought information about appropriate referral options, community-based support and psychological services would be helpful in ensuring longer term management. Finally, they also identified the negative attitudes of some senior clinicians and potential role models towards patients with MUS as a factor contributing to their own attitudes and management choices. Most reported a need for more training during the foundation years and recommended interactive case-based group discussions with a focus on providing meaningful explanations to patients for their symptoms. Case-based discussions and practical communication skills sessions were recommended as appropriate teaching methods, besides problem-based learning, the use of videos and roleplay with peers or simulated patients. Lecture-based teaching on the other hand was not recommended as it was considered that the topic of MUS requires an interactive approach [17].

Recommendations for improvement include addressing tutors’ negative attitudes and behavior towards functional syndromes, managing students’ exposure to patients with MUS or functional syndromes and identifying credible teachers and champions [10]. A short-cut strategy to improve consultations and management in this patient group would be to transfer knowledge and skills from reflexive and experienced GPs to students and junior doctors, enhancing a philosophy shift from curing to caring [18]. This multimodal approach could also include reliable coding methods to evaluate medical clinicians’ behavioral care skills in patients with MUS (Behavioral Health Treatment Model), to measure initial skills and improvement with training [19]. There is a general recognition that the treatment of illness behavior is most likely to be effective if it is integrated into the primary care medical context, where most patients present for care [3]. It has been demonstrated that care recommendations for structuring patient visits, providing reassurance and limiting unwarranted investigations, improve physical functioning but not necessarily the psychosocial distress that accompanies functional syndromes [3].

In a randomized control study two parallel groups of 39 patients and 156 patients were interviewed at baseline and at 3, 8 and 12 months after an intervention aiming to assess the effect of specific communication techniques on the self-perceived health of somatizing patients. In the intervention group (IG) GPs were trained in offering a physical explanatory framework and approaching sensitive topics in the patient’s experience indirectly. In the control (CG) group the GPs used the standard Goldberg reattribution technique [20]. Patients in both groups improved in all dimensions of the 36 item Medical Outcomes Study Short Form (MOS SF 36). The evolution over time of quality of life was, however, significantly better for the IG in five of the eight scales of the MOS SF-36, including bodily pain, mental health, physical functioning, vitality, and social functioning, and in the utility index [5, 21].

In a 2 level cluster randomized trial, GPs were randomized to receive training in reattribution techniques or non-specific psychosocial intervention to be applied to MUS patients. In the IG a 12 h specific training focused on specific skills necessary for the management of somatizing patients consisted of video feedback, role playing, video demonstrations, case discussions and modeling of desired behavior by the trainers. The CG relied on psychosocial primary care, an established component of standard GP postgraduate training in Germany. Multilevel modeling revealed a reduction of physical symptoms (*P* = 0.007), an improvement in physical functioning (*P* = 0.0172), and a reduction of depression (*P* = 0.0211) and anxiety (*P* = 0.0388) in the IG compared with the CG at the 3-month follow-up. However, results no longer remained significant after controlling for baseline and covariate variables besides a reduction of physical symptoms at 6-month follow-up (*P* = 0.029) [22].

A program for setting up an Intensive Short Term Dynamic Psychotherapy Service for MUS included teaching by MUS psychologists involved in university teaching programs for medical residents. The primary goal of this educational curriculum was to build skills in emotional awareness for self and patients. Various educational tools were integrated in the content areas and the learning objectives, including didactic seminaries with focus on patients or clinicians, group sessions with role play and other experiential exercises, videotaped physician/patient consultations, creating a library of learning resources and collaboration research with opportunities for elective placements. In this inclusive educational program patients reported significantly decreased somatic symptoms in the Patient Health Questionnaire-15 (*d* = 0.4). A statistically significant (23%) decrease in family physicians’ visits was found in the 6 months after attending the MUS service compared to the 6 months prior to the intervention. Both patients and primary care clinicians reported a high degree of satisfaction with the service [23].

The “train the trainer” principle was explored in the assessment of the hypothesis that a trained medical faculty can train residents effectively in a mental health care model. In a first step, medical faculty were trained in primary care mental health in a 15-month program; these trained educators subsequently taught internal medicine residents. The latter received approximately 75 h of predominantly experiential and comprised training in each of three training years. Significant improvements were documented in the IG, whereas the CG remained unchanged in pre- and post-test measurements for the primary endpoint variables of educating and informing, motivating, treatment statements, establishing commitment and goals, negotiating a treatment plan, using patient-centered non-emotional skills and, finally patient-centered emotional skills using a dichotomous coding procedure. Findings were similarly positive for models of patient-centered interviewing and informing and motivating. These improvements in mental health education seem highly relevant to MUS patients [24].

Weiland et al. described the stepwise development of an evidence-based post-graduate communication skills training program for medical specialists focused on patients with MUS to improve specialist interaction. A 14-h pilot training program was conducted in two groups for a total of 22 neurologists (both staff and residents), directed by two senior trainers. The training model was based on experiential learning and particularly focused on the improvement of exploration and information skills. Especially skills for symptom exploration, informing patients about the nature of MUS and effectively reassuring patients proved very useful. Knowledge about the epidemiology, etiology, and treatment of patients with MUS and somatoform disorders was selected as the first objective in the training program. Acquisition of skills in explanation such as informing patients about the nature of MUS and providing effective reassurance was the second major objective. Answering main patient concerns, paying attention to the somatic symptoms, sharing conclusions based on findings and using clinical experience are key elements of effective reassurance. Adequate reporting to the referring GP, containing the explanation and advice given to the patient about MUS (rather than limiting the approach, analysis, and reporting to the message that no explanations were found in the particular specialist domain) was defined as the third objective of the course. Structured learning techniques and Cumulative Micro Training were used in the design of the communication; techniques from Cognitive Behaviour Therapy (CBT) were adjusted to improve symptom exploration and explanation of MUS by medical specialists. Medical specialists and residents valued this evidence-based training program as highly relevant; they reported to profit from the acquired skills and experienced more satisfaction in their medical encounters with MUS patients [25].

In a multicenter RCT Weiland et al. assessed the effectiveness of this 14-h evidence-based communication training to improve specialists’ interviewing, information-giving skills in MUS consultations. An IG and a CG of medical specialists and co-assistants from 11 different specialties were compared using videotapes of MUS patients attending outpatient internal medicine (*n* = 193) or neurology (*n* = 94) clinics at baseline (*n* = 278) and at follow-up (*n* = 200). Education was performed mainly on secondary (60%) or tertiary (27%) level. The training, concerning exercises, skills, literature, duration and feedback was assessed as very useful for daily practice. There were indications that trained medical specialists had better interviewing and information-giving communication skills in MUS consultations. Nevertheless, medical specialists and residents still experienced consultations with MUS patients from different ethnic background as extremely difficult. The structure, which facilitated a more comfortable relationship with MUS patients, and the potential transfer of skills to a broader spectrum of patients with psychosocial problems were key elements valued by the specialists and medical residents [26].

In a multicenter cluster-randomized trial, the same research group evaluated the effects of a communication training for specialists on the quality of their reply letters to GPs about MUS patients. In an analysis of 478 MUS patients referred to 123 specialists, 80% of the physicians wrote at least one reply letter; in the assessment of 285 reply letters, trained physicians were more likely to report (61% vs 37%) and to answer (63 vs 33%) patient questions as compared to their untrained colleagues [27]. Training improved reply letters in addressing patient questions in contrast to GP’s referral questions, as is also documented in the mutual communication between specialists and GPs in general [28]. The issue of insufficiently reporting and answering GP’s referral questions may depend on the extent and the quality of GP referral letters to specialists [29]. Hence, likely both GPs and medical specialists need to be trained in writing appropriate referral and reply letters to improve health care to MUS patients. The reporting of somatic symptoms and of additional testing were well-developed among specialists with little room for improvement and are more likely to be part of classic training programs. In contrast, explanation of MUS with perpetuating factors in the bio-psychosocial model and the procurement of advice to patient and GP were only present in 27–41% and 54–69% of the reply letters, respectively, and hence remained areas for improvement. The authors advocated to discuss referral and reply letters about MUS patients with experts, to learn about ways of improving the exchange of valid information in MUS care at the interface between primary and secondary care [27].

In a position paper, Betancourt et al. described the contribution of cultural competence training to improving health outcomes and as one of the multiple strategies for addressing racial/ethnic disparities in health care [30]. Cultural competence education is considered by the Institute of Medicine a requirement for medical school and residency accreditation as well as for continuing medical education credits and medical licensure in some US states. Cultural competence training should be held to the same standards as other educational interventions and activities and should be evaluated in a stepwise fashion using the tools of health services research and the principles of quality improvement [30].

In a Delphi panel approach involving 34 experts from 11 countries a framework was developed of core cultural competencies (CC) for medical schoolteachers. A foremost key competency consists of knowledge on key concepts including culture and ethnicity, on how social and cultural factors can affect health, health-related behaviors, and health care and on key patient population groups to be identified in local sites (including but not limited to migration history, social conditions, specific health care needs, epidemiological data, and risk factors). A second key competency focuses on attitudes, defined as awareness of one’s implicit attitudes, including how personal norms, values and biases may affect health care provision and how culture shapes individual behavior and thinking, including the cultures of medicine. The third and final key competency identified relates to skills, defined as the ability to work effectively with an interpreter and identify and take into account socio-cultural factors that may influence patient care (e.g., provide a treatment plan that takes into account the patient’s social and cultural context). Cultural competence can be included in the curriculum in several ways, either as a specific core mandatory component or by ensuring CC is integrated into the curriculum wherever relevant. Success of integrating CC into the curriculum depends heavily on the support of those who develop institutional policies [31].

In a systematic review including 34 studies, excellent evidence was found that cultural competence training improves the knowledge of health care professionals (17 out of 19 studies in this domain demonstrating a beneficial effect) and good evidence that cultural competence training improves their attitudes and skills (beneficial effect in 21 of 25 studies evaluating attitudes and in all 14 studies evaluating skills). Moreover, all three studies assessing patient satisfaction turned out positive; however, no study at that time evaluated patient health status outcomes and a single study did not provide clear evidence for a positive impact on adherence [32].

## Discussion and recommendations

Current undergraduate training and education does not prepare medical students for diagnosing and managing patients with MUS, as evidenced in the study by Stenhoff et al. focusing on CFS/ME as a major syndrome within MUS. Educational interventions are needed to provide students with an acceptable and coherent model of CFS/ME and to give them the skills and confidence for diagnosis and management in their future medical practice. Training should not simply focus on students themselves, as opinions of senior doctors and medical establishment are frequently and through osmotic assimilation acquired by students, who perceive their trainers as authorities on medical knowledge, through a “hidden curriculum”, which includes negative attitudes from clinical staff [11]. “Teachers” often consider themselves insufficiently competent to deliver this specific training to students because of the lack of standardized training programs and insufficient clarity and explicitness of the required core competencies [30].

These shortcomings are related to the current medical culture strongly geared to biomedical thinking, stressing disease diagnosis and insufficiently focusing on illness and disability, which are prominent in MUS. Cultural and socioeconomic factors proved powerful predictors of individual somatic symptom perception and health care utilization in the domain of functional neurologic syndromes [33]. Dualistic health care systems with separation between somatic and mental health disciplines produce delayed diagnoses (with a mean estimated duration between onset of somatoform disorder and first psychotherapeutic and psychiatric treatment of 25 years) and increase stigma for mental disorders. They stress the need to include available and validated self-report instruments for screening and early diagnosis of functional disorders and somatic symptom disorders. With the aim to improve diagnosis, treatment and health care in patients with persistent somatic symptoms the European Network published recommendations for core outcome domains in the evaluation of interventions (EURONET–SOMA) [34]. Early recognition and treatment prevent unnecessary suffering and inappropriate health care utilization. The approach of functional disorders requires explanatory models for the pathway from symptom perception to functional syndromes. Access to effective diagnosis and treatment for all patients, accounting for cultural background, an emphasis on patient empowerment and early participation in the treatment process are key to outcome improvements. This implies enhancements in interdisciplinary training and collaboration between somatic and mental health disciplines.

It all starts at the roots of training health professionals. In spite of a lack of research into training students to diagnose and manage MUS, it needs to be acknowledged that there is a need to raise awareness about MUS during undergraduate teaching, with more formalized in-depth and clinically relevant, interactive teaching and training provided at times of greater clinical exposure such as during the foundation years or later during core medical training [35]. This should be based on own case experiences to encourage (inter)active group discussion [17]. Training should focus on communication techniques, particularly in relation to the communication of negative test results and specific examples of delivering physiological and psychological explanations for symptoms. Future interventions in the communication between trained GPs and their patients need to help patients to make sense of the complex nature of their problems. The aim is to reassure that medical attention to psychosocial factors does not preclude vigilance to physical disease and to establish a quality of relationship in which patients do not perceive psychosocial enquiry as inappropriate and thus create an environment in which physicians can support patient self-management [36]. Even after single and focused training programs, booster sessions will be required to retain long term teaching effects.

These possible interventions include evidence-based but understandable simplifications of biological mechanisms, such as pain sensitization in chronic pain, as well as the use of encompassing metaphors (“if the human being cannot talk, the body will talk” or “the body keeps the score”, applicable to a significant number of chronic pain/fibromyalgia/PTSD patients). These biological mechanisms nevertheless need to remain embedded in the broader conceptualization of illness within a bio-psychosocial framework.

At the next career level, there is an urgent need to improve postgraduate training on MUS and avoid overinvestigation, as current training does not equip junior physicians with the necessary knowledge and skills to effectively and confidently treat these patients whose prevalence and relevance are found in virtually all areas of medicine. Training needs to focus on practical skill development to increase clinical knowledge in areas, such as delivering suitable explanations, and to incorporate individual management strategies to help junior doctors tolerate the uncertainty and complexity associated with MUS [17]. Finally, it is recommended that patients with MUS are involved in the development of training and how it should be delivered [11].

Postgraduate education in MUS focused on knowledge and communication skills is both relevant and necessary in general as well as in specialist care [25]. A structured MUS focused communication training program significantly increases the interviewing and information-giving skills of medical specialists and residents [26]. This approach proved feasible and effective. It is recommended to incorporate these training models into postgraduate education for medical specialists and residents, who often encounter MUS patients. In view of the high prevalence of these functional syndromes across specialties, this training probably should be generic. Intervention studies in primary care have demonstrated that GP training in specific communication techniques on self-perceived health as well as in reattribution techniques or psychosocial intervention improved patient physical and mental quality of life, although high levels of health anxiety are likely to mitigate the positive effects of reattribution [20, 37]. Although cultural competence training improved knowledge, attitudes and skills in health care professionals, this translation into effective improvements of quality of life in MUS patients has not been documented yet [32]**.** Monitoring and evaluating patient-centered care for ethno-cultural communities allows for improvements in the delivery of culturally competent health care; future research should include development of patient-centered quality indicators for measuring cultural competence that also reflect cultural humility and the involvement of ethno-cultural communities in their development and implementation. This recent systematic review confirmed that the focus has rather been on structural and process than on outcome indicators [38].

Achieving a continuum of training is hence necessary to achieve a long term and lasting change in attitudes towards MUS patients. Senior or peer health professionals are significant role models and are key in shaping and altering trainees’ attitudes; hence, negative attitudes towards MUS patients are likely to be passed on to trainees. Attitude development through increased knowledge and skills needs to cut as transversally as possible through the whole of health care, and not remain limited to specialized units, as MUS presentations are likely and often prevalent in many health care settings. Attitudes of clinical tutors towards patients with MUS hence need to be addressed and rendered more positive, hopeful and evidence-based. The effectiveness of interventions aimed at improving attitude needs to be assessed in future research. This underscores that educational interventions to equip students to work effectively with patients presenting with functional syndromes/somatization/MUS needs to be supplemented by addressing the learning needs of medical educators and clinical tutors to have significant impact. This probably holds true even stronger for patients with a different ethnic backgrounds and migrants or refugees.

Indeed, increasing diversity due to migration and the presence of large groups of refugees from crisis areas leads to specific challenges, such as taking into account health competencies or “health literacy”, proper communication in spite of language barriers. Misunderstandings due to cultural differences in perceptions of illness/disease concepts and their treatment need to be avoided [38]. These challenges and barriers need to be recognized as well as acknowledged by caregivers and be approached in the correct manner to guarantee and enhance the delivery of high-quality care. This requires specific knowledge, attitudes, and skills. Medical teachers agree that this can only be achieved through specific training in the medical curriculum [31]. Specific training programs should be mandatory and aim at enhancing cultural competence both in health organizations and individual health professionals, including cultural competencies specific for the care of immigrant patients [38]. These programs may include training in working with interpreters and in other cultural competences to allow effective and safe practice in a multicultural society. Culturally competent health care settings include the provision of interpreters, clear policies, and procedures about how to use them effectively, and cultural competency education for health service providers that contains education on how culture (including their own) affects their institutions, practices and attitudes. This again needs to be included early in the training of health care professionals as well as throughout their professional careers in a philosophy of life-long learning. Cultural competence among health professionals indeed needs to be viewed as a strategy to ensure equal access to healthcare across diverse groups and to ensure that patients receive care in proportion and in accordance with their needs [31].

## Conclusion

Educational policy makers need research-based curricular information to guide a restructuring of medical education, that better aligns with societal needs. The reported interventional trials on feasible programs of communication and reporting teaching on MUS and on improved mental health education using the mental health care model as well as patient-centered interviewing, informing, and motivating, provide inspiration for such reforms of under- and postgraduate teaching [26]. There is a need for a competency framework that describes the knowledge, attitudes, and skills on (intercultural) communication and MUS to be acquired throughout the continuum of medical education.

## Data Availability

All data are available as electronic files upon request.

## References

[1] Dantzer R (2005). Somatization: a psychoneuroimmune perspective. Psychoneuroendocrinology.

[2] De Gucht V, Fischler B (2002). Somatization: a critical review of conceptual and methodological issues. Psychosomatics.

[3] Kirmayer LJ, Looper KJ (2006). Abnormal illness behaviour: physiological, psychological and social dimensions of coping with distress. Curr Opin Psychiatry.

[4] Rief W, Broadbent E (2007). Explaining medically unexplained symptoms-models and mechanisms. Clin Psychol Rev.

[5] Aiarzaguena JM, Grandes G, Gaminde I, Salazar A, Sánchez A, Ariño J (2007). A randomized controlled clinical trial of a psychosocial and communication intervention carried out by GPs for patients with medically unexplained symptoms. Psychol Med.

[6] Dobkin PL, De Civita M, Abrahamowicz M, Baron M, Bernatsky S (2006). Predictors of health status in women with fibromyalgia: a prospective study. Int J Behav Med.

[7] Collins JC, Rocco TS (2014). Disparities in healthcare for racial, ethnic, and sexual minorities. New Dir Adult Contin Educ.

[8] Page MJ, McKenzie JE, Bossuyt PM, Boutron I, Hoffmann TC, Mulrow CD (2021). Updating guidance for reporting systematic reviews: development of the PRISMA 2020 statement. J Clin Epidemiol.

[9] Hawker S, Payne S, Kerr C, Hardey M, Powell J (2002). Appraising the evidence: reviewing disparate data systematically. Qual Health Res.

[10] Joyce E, Cowing J, Lazarus C, Smith C, Zenzuck V, Peters S (2018). Training tomorrow’s doctors to explain ‘medically unexplained’physical symptoms: an examination of UK medical educators’ views of barriers and solutions. Patient Educ Couns.

[11] Stenhoff AL, Sadreddini S, Peters S, Wearden A (2015). Understanding medical students’ views of chronic fatigue syndrome: a qualitative study. J Health Psychol.

[12] Album D, Westin S (2008). Do diseases have a prestige hierarchy? A survey among physicians and medical students. Soc Sci Med.

[13] Vermeir P, Mariman A, Csabai M, Látos M, Weiland A, Stegers-Jager MK (2021). Perceptions and attitudes of health care givers and patients on medically unexplained symptoms: a narrative review with a focus on cultural diversity and migrants. Med Clin Res.

[14] Mariman A, Delesie L, Tobback E, Hanoulle I, Sermijn E, Vermeir P (2013). Undiagnosed and comorbid disorders in patients with presumed chronic fatigue syndrome. J Psychosom Res.

[15] Finset A (2018). Why do doctors not learn how to explain" medically unexplained symptoms"?. Patient Educ Couns.

[16] Asbring P, Närvänen AL (2003). Ideal versus reality: physicians perspectives on patients with chronic fatigue syndrome (CFS) and fibromyalgia. Soc Sci Med.

[17] Yon K, Nettleton S, Walters K, Lamahewa K, Buszewicz M (2015). Junior doctors’ experiences of managing patients with medically unexplained symptoms: a qualitative study. BMJ Open.

[18] Johansen M-L, Risor MB (2017). What is the problem with medically unexplained symptoms for GPs? A meta-synthesis of qualitative studies. Patient Educ Couns.

[19] Grayson-Sneed KA, Smith RC (2018). A research coding method to evaluate medical clinicians conduct of behavioral health care in patients with unexplained symptoms. Patient Educ Couns.

[20] Goldberg D, Gask L, O'Dowd T (1989). The treatment of somatization: teaching techniques of reattribution. J Psychosom Res.

[21] Brazier J, Roberts J, Deverill M (2002). The estimation of a preference-based measure of health from the SF-36. J Health Econ.

[22] Larisch A, Schweickhardt A, Wirsching M, Fritzsche K (2004). Psychosocial interventions for somatizing patients by the general practitioner: a randomized controlled trial. J Psychosom Res.

[23] Cooper A, Abbass A, Zed J, Bedford L, Sampalli T, Town J (2017). Implementing a psychotherapy service for medically unexplained symptoms in a primary care setting. J Clin Med.

[24] Smith RC, Laird-Fick H, Dwamena FC, Freilich L, Mavis B, Grayson-Sneed K (2018). Teaching residents mental health care. Patient Educ Couns.

[25] Weiland A, Blankenstein AH, Willems MHA, Van Saase JLCM, Van der Molen HT, Van Dulmen AM (2013). Post-graduate education for medical specialists focused on patients with medically unexplained physical symptoms; development of a communication skills training programme. Patient Educ Couns.

[26] Weiland A, Blankenstein AH, Van Saase JLCM, Van der Molen HT, Jacobs ME, Abels DC (2015). Training medical specialists to communicate better with patients with medically unexplained physical symptoms (MUPS). A randomized, controlled trial. PLoS ONE.

[27] Weiland A, Blankenstein AH, Willems MHA, Van Saase JLCM, Van Daele PLA, Van der Molen HT (2015). Training specialists to write appropriate reply letters to general practitioners about patients with medically unexplained physical symptoms; a cluster-randomized trial. Patient Educ Couns.

[28] Vermeir P, Vandijck D, Degroote S, Ommeslag D, Van De Putte M, Heytens S (2015). Mutual perception of communication between general practitioners and hospital-based specialists. Acta Clin Belg.

[29] Vermeir P, Vandijck D, Degroote S, Peleman R, Verhaeghe R, Mortier E (2015). Communication in healthcare: a narrative review of the literature and practical recommendations. Int J Clin Pract.

[30] Betancourt JR, Green AR (2010). Commentary: linking cultural competence training to improved health outcomes: perspectives from the field. Acad Med.

[31] Sorensen J, Norredam M, Dogra N, Essink-Bot M-L, Suurmond J, Krasnik A (2017). Enhancing cultural competence in medical education. Int J Med Educ.

[32] Beach MC, Price EG, Gary TL, Robinson KA, Gozu A, Palacio A (2005). Cultural competence: a systematic review of health care provider educational interventions. Med Care.

[33] Löwe B, Gerloff C (2018). Functional somatic symptoms across cultures: perceptual and health care issues. Psychosom Med.

[34] Rief W, Burton C, Frostholm L, Henningsen P, Kleinstäuber M, Kop WJ (2017). Core outcome domains for clinical trials on somatic symptom disorder, bodily distress disorder, and functional somatic syndromes: european network on somatic symptom disorders recommendations. Psychosom Med.

[35] Peters S, Rogers A, Salmon P, Gask L, Dowrick C, Towey M (2009). What do patients choose to tell their doctors? Qualitative analysis of potential barriers to reattributing medically unexplained symptoms. J Gen Intern Med.

[36] Blankenstein AH. Somatising patients in general practice: Reattribution, a promising approach. 2001. [PhD-Thesis - Research and graduation internal, Vrije Universiteit Amsterdam]

[37] Ahmed S, Siad FM, Manalili K, Lorenzetti DL, Barbosa T, Lantion V (2018). How to measure cultural competence when evaluating patient-centred care: a scoping review. BMJ Open.

[38] Suurmond J, Uiters E, de Bruijne MC, Stronks K, Essink-Bot M-L (2010). Explaining ethnic disparities in patient safety: a qualitative analysis. Am J Public Health.

